# Supplementary material for: Expanding global vaccine manufacturing capacity: Strategic prioritization in small countries
Source: PLOS Glob Public Health. 2023 Jun 29;3(6):e0002098. doi: 10.1371/journal.pgph.0002098 (PMC10309624; doi:10.1371/journal.pgph.0002098)
Supplement: S1 Text — (DOCX) [file pgph.0002098.s001.docx]

**Supporting Information**

**S1 Text. Methods**

We conducted an online search of peer-reviewed publications, websites, articles, and reports that reported vaccine manufacturing capacity in countries. The search strategy was designed to retrieve all publications, reports and articles that were publicly available as of April 30, 2022 and was limited neither by language, study design, type of publication, nor date of publication.

Identification of countries and companies with vaccine manufacturing capacity.

Appropriate search terms were used to extract 1) peer-reviewed publications from the electronic database PubMed, 2) information from international organizations, non-governmental organizations, vaccine manufacturing companies and institutions, news media and reports using Google Advanced Search. Furthermore, previously established data sources were also used to extract information on countries and companies with vaccine manufacturing capacity (Knowledge Ecology International. "KEI Notes on Vaccine Manufacturing Capacity" <https://www.keionline.org/covid-19-vaccine-manufacturing-capacity>). Refer to S1 Appendix, column “References and Sources” for a full list of sources with links.

The following search terms were used using appropriate AND/OR Boolean filters: [country name], “vaccine”, “COVID-19”, “manufacturing”, “capacity”.

Upon identification of companies with vaccine manufacturing capacity, we systematically extracted information on site location of manufacturing facilities and headquarters of the company. If the company had a manufacturing facility located in a country, regardless of whether the headquarters was in a different country, we assigned the country to have vaccine manufacturing capacity.

Identification of vaccines manufactured, steps of vaccine production and WHO prequalification.

To determine vaccine manufacturing portfolios of each company, we extracted information from the respective private pharmaceutical company or public institute webpage including the names of the vaccine manufactured and the stage of vaccine manufacturing service offered (Bioprocessing and Formulation, and/or Fill, Finish and Packaging). A country was only assigned to have ‘Bioprocessing and Formulation’, and/or ‘Fill, Finish and Packaging’ capacity if at least one company facility was documented to provide these services.

Next, we identified the vaccine manufacturing platform present in a country based on the vaccine manufacturing portfolios. The vaccine manufacturing platform for each company manufactured vaccine was identified, systematically documented, and classified as inactivated vaccines, live-attenuated vaccines, subunit vaccines, virus-like particle vaccines, viral-vector vaccines, and RNA based vaccines. A country was assigned to have a particular vaccine manufacturing platform, only if the company facility had a vaccine that used a respective vaccine manufacturing platform.

We also extracted WHO vaccine prequalification list from WHO’s prequalified vaccine website: https://extranet.who.int/pqweb/vaccines/prequalified-vaccines (Accessed April 1, 2022). Using this list, we were able to extract information on which countries had medical product regulatory authorities that had regulatory oversight of manufacturing facilities with prior/current prequalified vaccine.
