# Supplementary figures and images for: Expanding global vaccine manufacturing capacity: Strategic prioritization in small countries
Source: PLOS Glob Public Health. 2023 Jun 29;3(6):e0002098. doi: 10.1371/journal.pgph.0002098 (PMC10309624; doi:10.1371/journal.pgph.0002098)

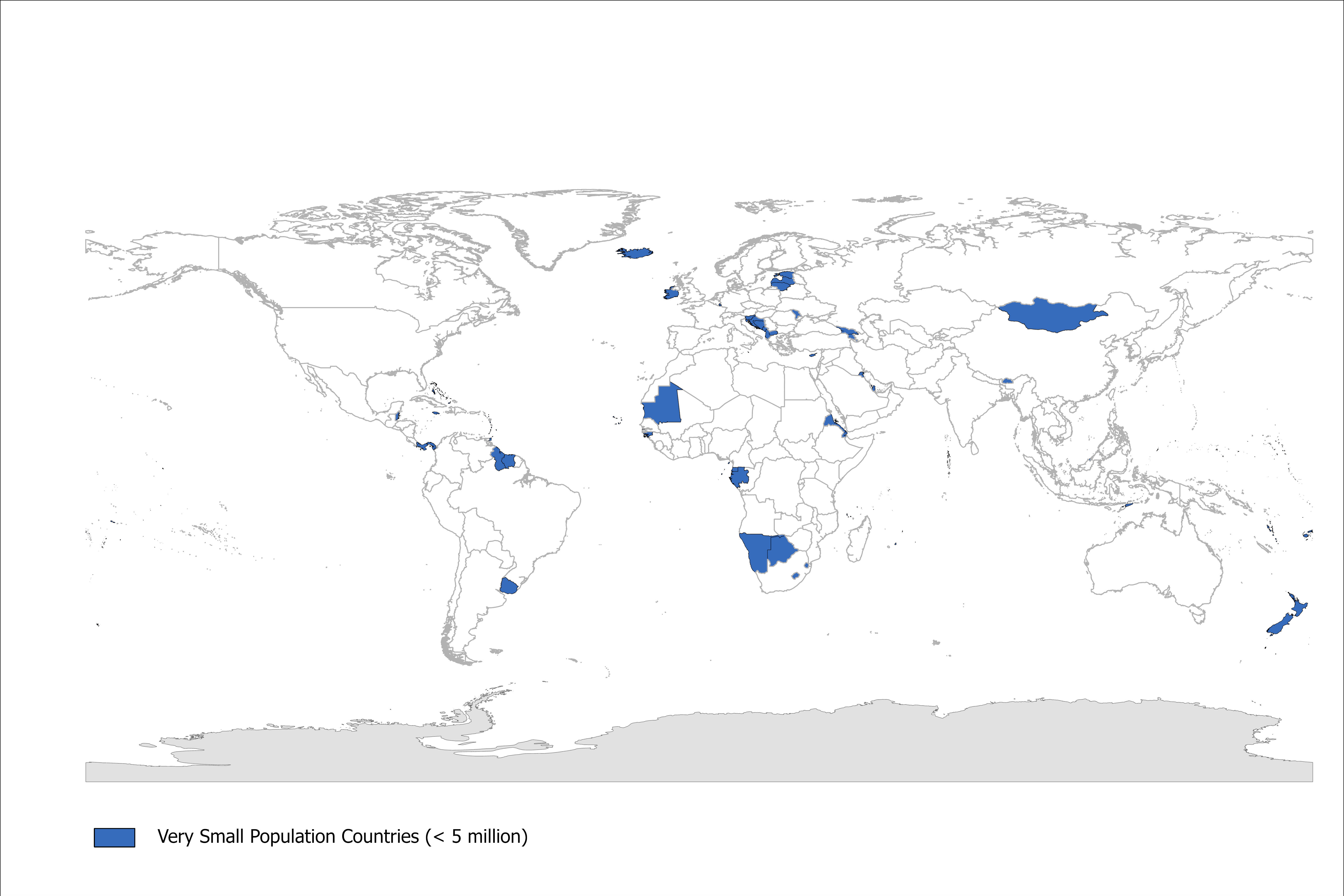

Supplement: S1 Fig — While these countries may not have existing vaccine manufacturing capacity or regulatory systems, countries from this list can be identified to potentially serve as vaccine manufacturing hubs, while ensuring geographical diversity. World Countries map package (Source: Esri Data and Maps) was used as the basemap [20]. The countries are: Albania, Andorra, Antigua and Barbuda, Armenia, Bahamas, Bahrain, Barbados, Belize, Bhutan, Bosnia and Herzegovina, Botswana, Brunei Darussalam, Cabo Verde, Comoros, Cook Islands, Croatia, Cyprus, Djibouti, Dominica, Equatorial Guinea, Eritrea, Estonia, Eswatini, Fiji, Gabon, Gambia, Georgia, Grenada, Guinea-Bissau, Guyana, Iceland, Ireland, Jamaica, Kiribati, Kuwait, Latvia, Lesotho, Lithuania, Luxembourg, Maldives, Malta, Marshall Islands, Mauritania, Mauritius, Federated States of Micronesia, Monaco, Mongolia, Montenegro, Namibia, Nauru, New Zealand, Niue, North Macedonia, Palau, Panama, Qatar, Republic of Moldova, Saint Kitts and Nevis, Saint Lucia, Saint Vincent and the Grenadines, Samoa, San Marino, Sao Tome and Principle, Seychelles, Slovenia, Solomon Islands, Suriname, Timor-Leste, Tonga, Trinidad and Tobago, Tuvalu, Uruguay, Vanuatu. (TIF) [file pgph.0002098.s002.tif]
