# Supplementary material for: Expanding global vaccine manufacturing capacity: Strategic prioritization in small countries
Source: PLOS Glob Public Health. 2023 Jun 29;3(6):e0002098. doi: 10.1371/journal.pgph.0002098 (PMC10309624; doi:10.1371/journal.pgph.0002098)
Supplement: S1 Table — (DOCX) [file pgph.0002098.s003.docx]

**Supporting Information**

**S1 Table. Detailed information on variables collected per country.**

| **Variable** | **Definition** |
| --- | --- |
| WHO region | Countries were classified into one of the six WHO regions (Eastern Mediterranean Region, European Region, African Region, Region of the Americas, Western Pacific Region, South-East Asia Region). |
| Determination of country vaccine production capacity | A country was defined as having the capacity for vaccine production if it contained at least one documented manufacturing facility with prior/current vaccine production activity. If a country was classified as No/Unknown, we were unable to find documented manufacturing facilities during our search. |
| Country population | The population of each country with vaccine manufacturing capacity (as of February 2022) was extracted from Worldometers (<https://www.worldometers.info/population/>) based on Worldometer elaboration of the United Nations data. The population of very small countries was extracted in July 2022. |
| Country vaccine portfolios | We assembled a list of private pharmaceutical companies, academic or public institutions involved in vaccine manufacturing per country using publicly available data sources. We also extracted information on the location of vaccine manufacturing facilities and company headquarters. Additionally, for each company, we extracted vaccines manufactured by disease. |
| Types of vaccines and manufacturing procedures | Vaccine manufacturing procedures are complex and require highly specialized technologies depending on the type of vaccine manufactured. Based on classification schemes previously described in the literature (<https://www.niaid.nih.gov/research/vaccine-types>, <https://www.nature.com/articles/s41563-020-0746-0>), we classified vaccines manufactured in each country as: inactivated vaccines, live-attenuated vaccines, subunit vaccines (including recombinant proteins, polysaccharides, toxoids, conjugate vaccines), virus-like particle vaccines, viral-vector vaccines, and RNA based vaccines (mRNA vaccines). Assessing existing vaccine manufacturing platforms in each country may provide insights into potential repurposing of existing platforms for expansion of COVID-19 vaccine production. |
| Steps of vaccine production | Since vaccine manufacturing is a complex and multifaceted procedure requiring multiple manufacturing steps, we also assembled information on whether countries have vaccine manufacturing facilities with ‘Bioprocessing and formulation’ capacity or ‘Fill, finish, and packaging’ capacity or both. If a country was classified as Unclear/Unknown, we were unable to find documentation of vaccine production steps during our search. |
| History of WHO prequalified vaccine production | We also extracted WHO vaccine prequalification list from WHO’s prequalified vaccine website: <https://extranet.who.int/pqweb/vaccines/prequalified-vaccines> (Accessed April 1, 2022). By prequalifying vaccines, WHO applies international standards to determine the safety and efficacy of vaccines which can then be used by UNICEF and other UN agencies for procurement of vaccines. Using this list, we were able to extract information on which countries had manufacturing facilities with prior/current prequalified vaccines. |
