# Supplementary material for: Expanding global vaccine manufacturing capacity: Strategic prioritization in small countries
Source: PLOS Glob Public Health. 2023 Jun 29;3(6):e0002098. doi: 10.1371/journal.pgph.0002098 (PMC10309624; doi:10.1371/journal.pgph.0002098)
Supplement: S2 Table — The list includes vaccine portfolios and vaccine manufacturing procedures in each country. (DOCX) [file pgph.0002098.s004.docx]

**Supporting Information**

**S2 Table. Global vaccine manufacturing capacity as of March 1, 2022.** The list includes vaccine portfolios and vaccine manufacturing procedures in each country.

| **Country*** | **WHO Region** | **Population** | **Vaccine Portfolio** | **Steps of Vaccine Production** | **Vaccine Manufacturing Platform** |
| --- | --- | --- | --- | --- | --- |
| Algeria | African Region | 45.1 million | COVID-19 | Bioprocessing and Formulation, Fill, Finish and Packaging | Inactivated Vaccine, Viral Vector Vaccine |
| Argentina | Region of the Americas | 45.8 million | COVID-19, Influenza, Pneumococcus, HPV, Hep A | Bioprocessing and Formulation, Fill, Finish and Packaging | Viral Vector Vaccine, Inactivated Vaccine, Subunit (recombinant protein, polysaccharide, toxoid, conjugate), Virus-like Particle |
| Australia | Western Pacific Region | 25.9 million | COVID-19, Influenza | Bioprocessing and Formulation, Fill, Finish and Packaging | Viral Vector Vaccine, Inactivated Vaccine, Subunit (recombinant protein, polysaccharide, toxoid, conjugate) |
| Austria | European Region | 9.0 million | COVID-19 | Bioprocessing and Formulation | RNA based (mRNA Vaccines) |
| Azerbaijan | European Region | 10.2 million | Influenza | Unclear | Inactivated Vaccine |
| Bangladesh | South-East Asia Region | 167.3 million | COVID-19, Cholera, Hep B, Influenza, Hep A, Measles, Rabies, Rubella, Typhoid Fever, Meningococcus, Tetanus | Bioprocessing and Formulation, Fill, Finish and Packaging | RNA based (mRNA Vaccines), Inactivated Vaccine, Subunit (recombinant protein, polysaccharide, toxoid, conjugate), Live-Attenuated Vaccine |
| Belgium | European Region | 11.6 million | COVID-19, HPV, Meningococcus, Diphtheria, Tetanus, Polio, Hep A, Hep B, Ebola, Pneumococcus | Bioprocessing and Formulation, Fill, Finish and Packaging | RNA based (mRNA Vaccines), Viral Vector Vaccine, Subunit (recombinant protein, polysaccharide, toxoid, conjugate), Virus-like Particle, Live-Attenuated Vaccine, Inactivated Vaccine |
| Brazil | Region of the Americas | 214.9 million | COVID-19, Yellow Fever, Polio, Meningococcus, Measles, Mumps, Rubella, *Haemophilus influenzae* Type B, Pneumococcus, Diphtheria, Influenza, Tetanus, Dengue, Hep B, Rabies, Tuberculosis, Rotavirus, Varicella | Bioprocessing and Formulation, Fill, Finish and Packaging | Viral Vector Vaccine, Inactivated Vaccine, Live-Attenuated Vaccine |
| Bulgaria | European Region | 6.8 million | Tuberculosis, Diphtheria, Tetanus | Unclear | Live-Attenuated Vaccine, Subunit (recombinant protein, polysaccharide, toxoid, conjugate) |
| Canada | Region of the Americas | 38.2 million | COVID-19, Diphtheria, Influenza, Pertussis, Tetanus, Tuberculosis, Polio | Bioprocessing and Formulation, Fill, Finish and Packaging | Subunit (recombinant protein, polysaccharide, toxoid, conjugate), Inactivated Vaccine, RNA based (mRNA Vaccines) |
| China | Western Pacific Region | 1.4 billion | COVID-19, Diphtheria, Tetanus, Pertussis, Influenza, Tuberculosis, *Haemophilus influenzae* Type B, Viral Hemorrhagic Fever, Japanese Encephalitis, Meningococcus, Measles, Mumps, Rubella, Polio, Rabies, Rotavirus, Varicella, Yellow Fever, HPV, Hep A | Bioprocessing and Formulation, Fill, Finish and Packaging | Inactivated Vaccine, Live-Attenuated Vaccine, Viral Vector Vaccine, Virus-like Particle |
| Colombia | Region of the Americas | 51.7 million | Tuberculosis, Influenza | Unclear | Live-Attenuated Vaccine |
| Cuba | Region of the Americas | 11.3 million | COVID-19, Tetanus, Diphtheria, Pertussis, Leptospirosis, Meningococcus, Typhoid Fever, Hep B | Bioprocessing and Formulation, Fill, Finish and Packaging | Subunit (recombinant protein, polysaccharide, toxoid, conjugate) |
| Czech Republic | European Region | 10.7 million | COVID-19 | Bioprocessing and Formulation | Subunit (recombinant protein, polysaccharide, toxoid, conjugate) |
| Denmark | European Region | 5.8 million | Tuberculosis, Ebola, Rabies, Smallpox, Polio | Bioprocessing and Formulation, Fill, Finish and Packaging | Viral Vector Vaccine, Live-Attenuated Vaccine, Inactivated Vaccine |
| Egypt | Eastern Mediterranean Region | 105.4 million | Cholera, Diphtheria, Tetanus, COVID-19, Influenza, Meningococcus, Typhoid Fever | Bioprocessing and Formulation, Fill, Finish and Packaging | Viral Vector Vaccine, Inactivated Vaccine, Subunit (recombinant protein, polysaccharide, toxoid, conjugate) |
| France | European Region | 65.5 million | Cholera, Diphtheria, Pertussis, Tetanus, *Haemophilus influenzae* Type B, Meningococcus, Tuberculosis, Typhoid Fever, Dengue, Hep A, Hep B, Influenza, Japanese Encephalitis, Polio, Rabies, Yellow Fever | Bioprocessing and Formulation, Fill, Finish and Packaging | Inactivated Vaccine, Subunit (recombinant protein, polysaccharide, toxoid, conjugate), Live-Attenuated Vaccine |
| Germany | European Region | 84.2 million | COVID-19, Influenza | Bioprocessing and Formulation, Fill, Finish and Packaging | RNA based (mRNA Vaccines), Inactivated Vaccine |
| India | South-East Asia Region | 1.4 billion | COVID-19, Diphtheria, Pertussis, Tetanus, Measles, Mumps, Rubella, *Haemophilus influenzae* Type B, Meningococcus, Influenza, Hep B, Polio, Tuberculosis, Rotavirus, Rabies, Typhoid Fever, Japanese Encephalitis, Pneumococcus | Bioprocessing and Formulation, Fill, Finish and Packaging | Live-Attenuated Vaccine, Inactivated Vaccine, Subunit (recombinant protein, polysaccharide, toxoid, conjugate), Viral Vector Vaccine |
| Indonesia | South-East Asia Region | 278.1 million | Diphtheria, Tetanus, Tuberculosis, *Haemophilus influenzae* Type B, Hep B, Polio, Measles, Japanese Encephalitis, COVID-19, Meningococcus, Varicella | Bioprocessing and Formulation, Fill, Finish and Packaging | Inactivated Vaccine, Subunit (recombinant protein, polysaccharide, toxoid, conjugate) |
| Iran, Islamic Rep. | Eastern Mediterranean Region | 85.7 million | Hep B, Tuberculosis, COVID-19 | Unclear | Inactivated Vaccine, Subunit (recombinant protein, polysaccharide, toxoid, conjugate) |
| Italy | European Region | 60.3 million | Meningococcus, Tetanus, Pertussis, Diphtheria, HPV, Hep B, *Haemophilus influenzae* Type B, Rabies, Rotavirus | Fill, Finish and Packaging | Viral Vector Vaccine, Virus-like Particle, Subunit (recombinant protein, polysaccharide, toxoid, conjugate), Inactivated Vaccine, Live-Attenuated Vaccine |
| Japan | Western Pacific Region | 125.8 million | Diphtheria, Tetanus, Influenza, Japanese Encephalitis, Hep B, Measles, Mumps, Rubella, Varicella, COVID-19, Tuberculosis | Bioprocessing and Formulation, Fill, Finish and Packaging | Inactivated Vaccine, Live-Attenuated Vaccine, Subunit (recombinant protein, polysaccharide, toxoid, conjugate) |
| Kazakhstan | European Region | 19.1 million | COVID-19 | Unclear | Viral Vector Vaccine |
| Mexico | Region of the Americas | 131.1 million | COVID-19, Diphtheria, Polio, Tetanus, Influenza | Bioprocessing and Formulation, Fill, Finish and Packaging | Viral Vector Vaccine, Inactivated Vaccine |
| Morocco | Eastern Mediterranean Region | 37.6 million | COVID-19 | Fill, Finish and Packaging | Viral Vector Vaccine, Inactivated Vaccine |
| Netherlands | European Region | 17.1 million | Diphtheria, Tetanus, Polio, Influenza, COVID-19 | Bioprocessing and Formulation, Fill, Finish and Packaging | Inactivated Vaccine, Virus-like Particle, Viral Vector Vaccine |
| Pakistan | Eastern Mediterranean Region | 227.7 million | COVID-19, Measles, Rabies, Cholera, Typhoid Fever, Tetanus | Bioprocessing and Formulation, Fill, Finish and Packaging | Subunit (recombinant protein, polysaccharide, toxoid, conjugate), Viral Vector Vaccine |
| Philippines | Western Pacific Region | 111.9 million | COVID-19 | Fill, Finish and Packaging | Subunit (recombinant protein, polysaccharide, toxoid, conjugate) |
| Republic of Korea | Western Pacific Region | 51.3 million | Cholera, Diphtheria, Tetanus, Hep B, Polio, Influenza, Varicella | Bioprocessing and Formulation, Fill, Finish and Packaging | Subunit (recombinant protein, polysaccharide, toxoid, conjugate), Inactivated Vaccine, Live-Attenuated Vaccine |
| Russian Federation | European Region | 146.0 million | COVID-19, Ebola, HPV, Rotavirus, Pneumococcus, Yellow Fever | Bioprocessing and Formulation, Fill, Finish and Packaging | Inactivated Vaccine, Viral Vector Vaccine, Subunit (recombinant protein, polysaccharide, toxoid, conjugate), Live-Attenuated Vaccine |
| Senegal | African Region | 17.4 million | Yellow Fever | Bioprocessing and Formulation, Fill, Finish and Packaging | Live-Attenuated Vaccine |
| Serbia | European Region | 8.6 million | Tuberculosis, Diphtheria, Tetanus, Influenza | Unclear | Live-Attenuated Vaccine, Subunit (recombinant protein, polysaccharide, toxoid, conjugate), Inactivated Vaccine |
| Singapore | Western Pacific Region | 5.9 million | Pneumococcus, *Haemophilus influenzae* Type B | Bioprocessing and Formulation | Subunit (recombinant protein, polysaccharide, toxoid, conjugate) |
| South Africa | African Region | 60.5 million | COVID-19, Tuberculosis, Measles, Pneumococcus, Hep B, Diphtheria, Tetanus, Polio, *Haemophilus influenzae* Type B | Fill, Finish and Packaging | RNA based (mRNA Vaccines), Viral Vector Vaccine |
| Sweden | European Region | 10.2 million | Influenza, Japanese Encephalitis, Cholera | Bioprocessing and Formulation | Inactivated Vaccine |
| Switzerland | European Region | 8.7 million | COVID-19 | Bioprocessing and Formulation | RNA based (mRNA Vaccines) |
| Thailand | South-East Asia Region | 70.0 million | Pertussis, Tuberculosis, Rabies, Diphtheria, Tetanus, COVID-19 | Bioprocessing and Formulation, Fill, Finish and Packaging | Viral Vector Vaccine, Live-Attenuated Vaccine |
| Tunisia | Eastern Mediterranean Region | 12.0 million | Rabies, Tuberculosis | Bioprocessing and Formulation, Fill, Finish and Packaging | Live-Attenuated Vaccine |
| Turkey | European Region | 85.8 million | COVID-19 | Unclear | Viral Vector Vaccine |
| United Kingdom of Great Britain and Northern Ireland | European Region | 68.4 million | Meningococcus, Tetanus, Pertussis, Diphtheria, HPV, Hep B, Hep A, Influenza, *Haemophilus influenzae* Type B, Rabies, Rotavirus, Cholera, Measles, Mumps, Rubella | Bioprocessing and Formulation, Fill, Finish and Packaging | Live-Attenuated Vaccine, Viral Vector Vaccine, Subunit (recombinant protein, polysaccharide, toxoid, conjugate) |
| United States of America | Region of the Americas | 334.1 million | Meningococcus, Pneumococcus, Tuberculosis, HPV, *Haemophilus influenzae* Type B, Measles, Mumps, Rubella, Hep A, Hep B, Rotavirus, Varicella, COVID-19, Influenza, Ebola | Bioprocessing and Formulation, Fill, Finish and Packaging | RNA based (mRNA Vaccines), Inactivated Vaccine, Subunit (recombinant protein, polysaccharide, toxoid, conjugate), Viral Vector Vaccine, Live-Attenuated Vaccine, Virus-like Particle |
| Vietnam | Western Pacific Region | 98.7 million | Diphtheria, Pertussis, Tetanus, Tuberculosis, Influenza, COVID-19 | Fill, Finish and Packaging | Inactivated Vaccine, Viral Vector Vaccine |

* A country was defined as having the capacity for vaccine production if it contained at least one documented manufacturing facility with prior/current vaccine production activity. If a country was classified as No/Unknown, we were unable to find documented manufacturing facilities during our search.
