# Supplementary material for: Expanding global vaccine manufacturing capacity: Strategic prioritization in small countries
Source: PLOS Glob Public Health. 2023 Jun 29;3(6):e0002098. doi: 10.1371/journal.pgph.0002098 (PMC10309624; doi:10.1371/journal.pgph.0002098)
Supplement: S3 Table — The vaccine manufacturing platforms used for their production and the countries producing these vaccines are included in this list. (DOCX) [file pgph.0002098.s005.docx]

**Supporting Information**

**S3 Table. List of COVID-19 vaccines manufactured by the 43 countries identified in our study as of March 1, 2022.** The vaccine manufacturing platforms used for their production and the countries producing these vaccines are included in this list.

| **COVID-19 Vaccine** | **Manufacturing Countries** | **Vaccine Manufacturing Platform** |
| --- | --- | --- |
| Sputnik V | Algeria, Argentina, Brazil, Egypt, India, Kazakhstan, Mexico, Morocco, Russia, Turkey, Vietnam | Viral Vector Vaccine |
| Sinovac-CoronaVac COVID-19 vaccine | Algeria, Brazil, China, Egypt, Indonesia | Inactivated Vaccine |
| Oxford/AstraZeneca (ChAdOx1-S [recombinant]) vaccine | Argentina, Australia, Belgium, India (CoviShield), Italy, Mexico, Thailand, UK | Viral Vector Vaccine |
| CureVac – CvnCoV COVID-19 vaccine (Terminated) | Austria | RNA-based (mRNA) Vaccine |
| Bangavax COVID-19 vaccine (in trials) | Bangladesh | RNA-based (mRNA) Vaccine |
| Pfizer-BioNTech BNT162b2 COVID-19 vaccine | Belgium, Germany, South Africa, Switzerland, USA | RNA-based (mRNA) Vaccine |
| Janssen Ad26.COV2.S COVID-19 vaccine | Netherlands, South Africa (Aspenovax), USA | Viral Vector Vaccine |
| Novavax NVX- CoV2373 vaccine | Canada, Czech Republic, India (Covovax), Japan, UK | Subunit Vaccine |
| PTX-COVID19 B mRNA vaccine (in trials) | Canada | RNA-based (mRNA) Vaccine |
| Sinopharm COVID-19 (BBIBP-CorV, COVILO) | China, Morocco | Inactivated Vaccine |
| Soberana 2 FINLAY-FR-2 vaccine | Cuba, Iran (Pasteurcovac) | Subunit Vaccine |
| Abdala CIGB-66 vaccine | Cuba | Subunit Vaccine |
| Bharat Biotech BBV152 Covaxin | India | Inactivated Vaccine |
| COVIran Barekat | Iran | Inactivated Vaccine |
| CanSino- Convidecia (Ad5-nCoV) COVID-19 Vaccine | China, Mexico, Pakistan (PakVac) | Viral-Vector Virus |
| AdaptVac ABNCoV2 COVID-19 vaccine (in trials) | Netherlands | Virus-like particle |
| EuCorVac-19 (in trials) | Philippines, South Korea | Subunit Vaccine |
| Moderna mRNA-1273 vaccine | Switzerland, USA | RNA-based (mRNA) Vaccine |
| LUNAR-COV19/ARCT-021 - Arcturus mRNA vaccine (in trials) | USA | RNA-based (mRNA) Vaccine |

Note - This list is not a full representation of the complete list of approved/authorized COVID-19 vaccines and candidate COVID-19 vaccines currently in development. Due to the fluid nature of vaccine approvals, authorizations, and those in clinical trials, and due to our online search strategy, it is possible that some vaccines and the countries producing these vaccines may have been missed during our online search. Additionally, some vaccines are in parenthesis in the “manufacturing countries” column as they have been assigned specific names in respective countries.
