# Supplementary material for: Expanding global vaccine manufacturing capacity: Strategic prioritization in small countries
Source: PLOS Glob Public Health. 2023 Jun 29;3(6):e0002098. doi: 10.1371/journal.pgph.0002098 (PMC10309624; doi:10.1371/journal.pgph.0002098)
Supplement: Attachment — Submitted filename: Response_to_Reviewers.docx [file pgph.0002098.s007.docx]

**Response to Reviewers**

We are grateful for the thorough review of our manuscript and for the opportunity to submit a revision for consideration. We have addressed the comments provided by the Reviewers below and have revised the manuscript accordingly.

**Journal Requirements:**

1. Please send a completed 'Competing Interests' statement, including any COIs declared by your co-authors. If you have no competing interests to declare, please state "The authors have declared that no competing interests exist". Otherwise please declare all competing interests beginning with the statement "I have read the journal's policy and the authors of this manuscript have the following competing interests:"

***Response***: We have included a ‘Competing Interests’ statement in the manuscript. Please see lines 439-440.

2. Please provide separate figure files in .tif or .eps format.

***Response***: We have uploaded figure files in the .tif format.

3. Please review your reference list to ensure that it is complete and correct. If you have cited papers that have been retracted, please include the rationale for doing so in the manuscript text, or remove these references and replace them with relevant current references. Any changes to the reference list should be mentioned in the rebuttal letter that accompanies your revised manuscript. If you need to cite a retracted article, indicate the article’s retracted status in the References list and also include a citation and full reference for the retraction notice.

***Response***: Thank you for bringing this to our attention. We have updated the references accordingly. In situations where the weblink is no longer available as websites may have been updated or removed since we completed this analysis (April 2022), we have updated references or indicated the article/website’s retracted status. Please see below for all updates.

Main Manuscript References:

In addition to adding new references in response to reviewer comments, we have updated the following three references to include complete information:

21. Mola EL, Acevedo BE, Silva R, Tormo B, Montero R, Herrera L. Development of Cuban biotechnology. J Commer Biotechnol. 2003 Jan 1;9(2): 147–152

30. Prabhala A, Alsalhani A. Pharmaceutical manufacturers across Asia, Africa and Latin America with the technical requirements and quality standards to manufacture mRNA vaccines. [Internet]. 2020 [cited 2022 Jul 20]. Available from: <https://msfaccess.org/pharmaceutical-firms-across-asia-africa-and-latin-america-potential-manufacture-mrna-vaccines>

39. Casey CA, Cimino-Isaacs CD. Congressional Research Service: Export Restrictions in Response to the COVID-19 Pandemic. [Internet]. 2021 [cited 2022 Jul 20]. Available from: <https://crsreports.congress.gov/product/pdf/IF/IF11551>

S1 Appendix:

| **Original Reference** | **Updated Reference** |
| --- | --- |
| ***Sheet 1: Countries w Vax Manufac Cap*** | |
| 2. CSL (2020). "Local Manufacturing of COVID-19 Vaccine Reaches Final Stages" https://www.csl.com/news/2021/20210212-local-manufacturing-of-covid-19-vaccine-reaches-final-stages | 2. CSL (2020). "Local Manufacturing of COVID-19 Vaccine Reaches Final Stages" https://www.cslseqirus.com.au/news/local-manufacturing-of-covid-19-vaccine-reaches-final-stages |
| 1. Sanofi in Azerbaijan. https://www.sanofi.az/en/our-products | 1. Sanofi in Azerbaijan. <https://www.sanofi.az/en/our-products> (webpage no longer available)  2. List of Pharmaceutical companies in Azerbaijan. https://www.fastbase.com/countryindex/Azerbaijan/P/Pharmaceutical |
| 5. Bio-Manguinhos. "Partnerships for the World Public Health" https://www.bio.fiocruz.br/en/images/stories/pdfs/folders-ingles/fol der-bio-international.pdf | 5. Bio-Manguinhos. "World Presence" https://www.bio.fiocruz.br/index.php/en-us/about-us-bio/world-presence |
| 10. Innovax. http://www.innovax.cn/en/about.aspx | 10. Innovax .https://www.ystwt.cn/category/innovax/ |
| 7. PAHO (2002). "Development of Cuban biotechnology" <https://www.paho.org/cub/dmdocuments/BIOLopezEetal.pdf> | 7. PAHO (2003). "A first hand report on Cuban biotechnology" https://www3.paho.org/cub/dmdocuments/BIOBakerGCuban_biotechnology.pdf |
| 4. Statens Serum Institut. Vaccine Development. https://en.ssi.dk/about-us/contact/departments/v/vaccine-development | 4. Statens Serum Institut. Vaccine Development. <https://en.ssi.dk/about-us/contact/departments/v/vaccine-development> (webpage no longer available)  10. Statens Serum Institute. https://www.tbvi.eu/staten-serum-institute/ |
| 5. Statens Serum Institut. Contract GMP/GCP Services. https://en.ssi.dk/products-and-services/contract-gmp-gcp-services | 5. Statens Serum Institut. Contract GMP/GCP Services. https://en.ssi.dk/products-and-services/contract-gmp-gcp-services (webpage no longer available)  10. Statens Serum Institute. https://www.tbvi.eu/staten-serum-institute/ |
| 2. DCVMN. Biofarma. https://www.dcvmn.org/_Bio-Farma_ | 2. DCVMN. Biofarma. https://dcvmn.org/member/biofarma/ |
| 4. Avara Pharmaceutical Services (2020). "Avara Liscate Facility Welcomes 2020 On a High!" https://www.avara.com/events-and-media/avara-liscate-facility-welcomes-2020-on-a-high | 4. Avara Pharmaceutical Services (2020). "Avara Liscate Facility Welcomes 2020 On a High!" https://www.aiac.com/news/2019/3/11/monmouth-glacier-ak-e7j2m-szwsh |
| 2. USAID (2021). Ensuring Kazakhstan's Uninterrupted Access to Safe Medicines. https://www.usaid.gov/kazakhstan/success-stories/sep-2021-ensuring-kazakhstan-uninterrupted-access-safe-medicines | 2. USAID (2021). Ensuring Kazakhstan's Uninterrupted Access to Safe Medicines. https://www.usaid.gov/kazakhstan/news/ensuring-kazakhstans-uninterrupted-access-safe-medicines |
| 2. Morocco Embassy. "Manufacturing of anti-COVID-19 in Morocco" http://www.moroccoembassy.org.au/?q=news/manufacturing-anti-covid-19-morocco | 2. Morocco Embassy. "Morocco to Start Manufacturing COVID-19 Vaccine" https://www.moroccoembassy.co.za/morocco-in-the-news/484-morocco-to-start-manufacturing-covid-19-vaccine |
| 6. DCVMN. Vaccine Manufacturing in Africa. https://www.dcvmn.org/IMG/pdf/20210316_vx_mf_africa_dcvmn_briefing_vpre-read.pdf | 6. DCVMN. Vaccine Manufacturing in Africa. <https://www.dcvmn.org/IMG/pdf/20210316_vx_mf_africa_dcvmn_briefing_vpre-read.pdf> (webpage no longer available) |
| 1. National Institute of Health - Islamic Republic of Pakistan. Biological Product Division. https://www.nih.org.pk/biological-production-division/ | 1. National Institute of Health - Islamic Republic of Pakistan. Vaccine and Biological Products Center. https://www.nih.org.pk/vaccine-and-biological-products-center |
| 3. National Institute of Health - Islamic Republic of Pakistan. Viral Vaccines. https://www.nih.org.pk/type/viral-vaccines/ | 3. National Institute of Health - Islamic Republic of Pakistan. Viral Vaccines. https://www.nih.org.pk/type/viral-vaccines/ (webpage not available) |
| ***Sheet 2: Companies w Vax Manufac Cap*** | |
| 3. DCVMN AGM 2021. https://www.dcvmn.org/IMG/pdf/d1_s3_5_sinergiumbiotech_oct2021_dcvmn.pdf | 3. DCVMN AGM 2021. https://dcvmn.org/wp-content/uploads/2021/12/d1_s3_5_sinergiumbiotech_oct2021_dcvmn.pdf |
| 5. Company website: https://www.richmondlab.com/ar/en/inicio-en/ | 5. Company website: https://richmondlab.com/ar/ |
| 1. Sanofi in Azerbaijan. Our Products. https://www.sanofi.az/en/our-products | 1. Sanofi in Azerbaijan. Our Products. https://www.sanofi.az/en/our-products (webpage no longer available) |
| 1. Bio-Manguinhos. "Partnerships for the World Public Health" https://www.bio.fiocruz.br/en/images/stories/pdfs/folders-ingles/fol der-bio-international.pdf | 1. Bio-Manguinhos. "World Presence" https://www.bio.fiocruz.br/index.php/en-us/about-us-bio/world-presence |
| 4. PAHO (2002). "Development of Cuban biotechnology" <https://www.paho.org/cub/dmdocuments/BIOLopezEetal.pdf> | 4. PAHO (2003). "A first hand report on Cuban biotechnology" https://www3.paho.org/cub/dmdocuments/BIOBakerGCuban_biotechnology.pdf |
| 1. Statens Serum Institut. Vaccine Development. https://en.ssi.dk/about-us/contact/departments/v/vaccine-development | 1. Statens Serum Institut. Vaccine Development. <https://en.ssi.dk/about-us/contact/departments/v/vaccine-development> (webpage no longer available)  4. Statens Serum Institute. https://www.tbvi.eu/staten-serum-institute/ |
| 2. Statens Serum Institut. Contract GMP/GCP Services. https://en.ssi.dk/products-and-services/contract-gmp-gcp-services | 2. Statens Serum Institut. Contract GMP/GCP Services. https://en.ssi.dk/products-and-services/contract-gmp-gcp-services (webpage no longer available)  4. Statens Serum Institute. https://www.tbvi.eu/staten-serum-institute/ |
| 1. DCVMN. Biofarma. https://www.dcvmn.org/_Bio-Farma_ | 1. DCVMN. Biofarma. https://dcvmn.org/member/biofarma/ |
| 2. Avara Pharmaceutical Services (2020). "Avara Liscate Facility Welcomes 2020 On a High!" https://www.avara.com/events-and-media/avara-liscate-facility-welcomes-2020-on-a-high | 2. Avara Pharmaceutical Services (2020). "Avara Liscate Facility Welcomes 2020 On a High!" https://www.aiac.com/news/2019/3/11/monmouth-glacier-ak-e7j2m-szwsh |
| 2. USAID (2021). Ensuring Kazakhstan's Uninterrupted Access to Safe Medicines. https://www.usaid.gov/kazakhstan/success-stories/sep-2021-ensuring-kazakhstan-uninterrupted-access-safe-medicines | 2. USAID (2021). Ensuring Kazakhstan's Uninterrupted Access to Safe Medicines. https://www.usaid.gov/kazakhstan/news/ensuring-kazakhstans-uninterrupted-access-safe-medicines |
| 1. Landsteiner Scientific, laboratorio farmacéutico. https://www.landsteiner.com/en/laboratory/ | 1. Landsteiner Scientific, laboratorio farmacéutico. <https://www.landsteiner.com/en/laboratory/> (webpage not loading) |
| 2. Morocco Embassy. "Manufacturing of anti-COVID-19 in Morocco" http://www.moroccoembassy.org.au/?q=news/manufacturing-anti-covid-19-morocco | 2. Morocco Embassy. "Morocco to Start Manufacturing COVID-19 Vaccine" https://www.moroccoembassy.co.za/morocco-in-the-news/484-morocco-to-start-manufacturing-covid-19-vaccine |
| 1. Tony Blair Institute for Global Change. "Vaccine Manufacturing in Africa: What It Takes and Why It Matters" https://institute.global/sites/default/files/articles/Vaccine-Manufacturing-in-Africa-What-It-Takes-and-Why-It-Matters.pdf | 1. Tony Blair Institute for Global Change. "Vaccine Manufacturing in Africa: What It Takes and Why It Matters" https://www.institute.global/insights/public-services/vaccine-manufacturing-africa-what-it-takes-and-why-it-matters |
| 6. DCVMN. Vaccine Manufacturing in Africa. https://www.dcvmn.org/IMG/pdf/20210316_vx_mf_africa_dcvmn_briefing_vpre-read.pdf | 6. DCVMN. Vaccine Manufacturing in Africa. <https://www.dcvmn.org/IMG/pdf/20210316_vx_mf_africa_dcvmn_briefing_vpre-read.pdf> (webpage no longer available) |
| 2. National Institute of Health - Islamic Republic of Pakistan. Biological Product Division. https://www.nih.org.pk/biological-production-division/ | 2. National Institute of Health - Islamic Republic of Pakistan. Vaccine and Biological Products Center. https://www.nih.org.pk/vaccine-and-biological-products-center |
| 3. National Institute of Health - Islamic Republic of Pakistan. Viral Vaccines. https://www.nih.org.pk/type/viral-vaccines/ | 3. National Institute of Health - Islamic Republic of Pakistan. Viral Vaccines. https://www.nih.org.pk/type/viral-vaccines/ (webpage not available) |
| 1. Innovax. http://www.innovax.cn/en/about.aspx | 1. Innovax .https://www.ystwt.cn/category/innovax/ |

**Reviewer #1 (Comments for the Author)**:

The authors have painstakingly addressed the issue of vaccine accessibility disparity that had existed between the first world and others for a long time which was made more visible during the COVID-19 pandemic. The design and the methodical approach at identifying low population countries with capacity and capability that could be supported technically to manufacture vaccines either singly or in collaboration with other entities seem to be reasonable to reduce the vaccine nationalism that was clearly demonstrated during the COVID pandemic. The need for strengthening these manufacturing facilities and their corresponding regulatory authorities for prequalification purposes were also emphasized. Africa has been in the eye of the storm and has been disproportionally affected during this period. With a huge population, poor per capita income and low capacity for production of molecular-derived vaccines e.g. mRNA, attention should be centered on how this region and allied regions could be assisted. Overall, this manuscript has highlighted the weaknesses of the strong vaccine producing nations to equitably protect the weaker ones during pandemic and has put up a lot of salient recommendations and suggestions on how the future pandemic could be approached based on the current experience.

***Response***: We would like to sincerely thank Reviewer 1 for their comments regarding the manuscript. We appreciate their feedback and look forward to submitting a revised version of the manuscript.

**Reviewer #2 (Comments for the Author)**:

This is an interesting paper and contribution to the lit on policy proposals for address equity in the next pandemic. Congratulations to the authors, and my apologies for the week’s delay in my review – I was very ill.

This is a good draft, but there remain some points in the argument that could be strengthened.

***Response***: Thank you very much for your comments. We really appreciate your feedback and discussion points, and we look forward to submitting a revised version of the manuscript with your suggestions.

The introduction effectively reviews the failure of global vaccine distribution. The methods/results show where existing capacity is, with more detailed analysis for small/very small countries and prequal capacity. The argument that manufacturing capacity could be concentrated in small countries to mitigate vaccine nationalism is argued/theoretical.

The main weakness in the paper is that it does not effectively address counterarguments or limitations. The idea of concentrating manufacturing capacity in small countries is novel /contrary to the accepted wisdom that investments in manufacturing capacity should prioritize a) low income countries, b) countries in regions with less capacity, and c) countries that are large enough to maintain markets in non-pandemic times. The countries identified by the authors as the best candidates would rank low across all of these criteria. It’s great that the authors are injecting new ideas into this space, but give how far the findings are (ie including small, rich European countries as priority candidates), it should engage more with the existing lit / policy discussions and pre-empt counterarguments.

***Response***: We agree with Reviewer 2 that a major strategy to improve vaccine access globally is to improve vaccine manufacturing and regulatory capacity in low-and middle-income countries. We believe that to improve vaccine access and equity, a whole range of strategies are needed, including both short-term and long-term strategies. Improving vaccine manufacturing capacity and building functional and mature regulatory systems for medical products, especially in LMICs, would likely fall under long-term strategies, given the significant time and investment required to build infrastructure, train workforce, and create financing arrangements. To this end, the strategy of diversifying global vaccine procurement by identifying small population countries with current vaccine manufacturing capacity and functional regulatory systems is a short-term strategy to combat the effects of vaccine nationalism which significantly hampered vaccine availability to facilities like COVAX. We have addressed your comments in the Discussion section, please see lines 397-424.

Some questions I had in reading the draft:

-How might excess capacity be financed in small countries? How would this capacity be used in not-pandemic times?

I see the argument for small countries exhausting domestic demand, but domestic foreign policy / trade policy is still very much on the table. We can’t assume that the exhaustion of demand would lead countries to be willing to work with international efforts / market mechanisms.

***Response***: Thank you for bringing up this point. To address this, we have included lines 407-413 in the Discussion.

-The elephant in the room is trade agreements/blocs/sanctions. At the center of the Venn diagram of prioritized countries (small, manufacturing capacity), history of prequal) are 5 countries: 4 in the European Union, and 1 under trade sanctions in much of the world. The former would still be subject to EU-level export controls until EU-wide demand was fulfilled, and the latter would be (and indeed, during the pandemic was) hampered in manufacturing and distribution but trade sanctions. This also links to financing… if we’re arguing for more-than-national capacity, one would expect some level of investment (private or public), and prioritizing rich European countries that did not deliver in the last pandemic is, for me, a bit of a hard sell.

***Response***: We agree with Reviewer 2 that these are major hurdles and excellent discussion points to consider when thinking of strategies to improve vaccine access, globally. To improve vaccine access and equity, a comprehensive policy toolkit is required that utilizes multiple strategies to improve availability of medical products. Diversifying and procuring vaccines from many sources, including small countries, for facilities like COVAX is just one of the many strategies that policymakers could consider employing for future health emergencies.

Regarding EU wide export controls during the COVID-19 pandemic, EU’s major policy position to respond to future pandemics is limiting the use of export restrictions in addition to the use of licensing agreements. Indeed, they have highlighted that vaccine producing countries “should be ready to export a fair share of their domestic production. In addition, the EU considers that supplies to the COVAX Facility should never be restricted, and no measures should limit trade in inputs necessary for the production of COVID-19 vaccines and treatments.” This provides an opportunity for the Pandemic Treaty to negotiate obligations from such vaccine producing countries, including small vaccine manufacturing countries, to aid in efforts to improve vaccine access. Additionally, although Cuba does have trade sanctions, it has exported vaccines to more than 40 countries and has a history of producing vaccines being WHO prequalified and procured by UN agencies.

To address these discussion points and limitations, we have included lines 397-424 in the Discussion section.

Contribution to the lit- sell your results! Is this the first compilation of global vaccine manufacturing capacity by country? Say so! If not, explain how it fits in with existing databases / papers.

***Response***: Thank you for your comment. We agree that this point is crucial to the novelty of our paper and have included this point in lines 90-93.

Relation to the lit – worth briefly outlining the lit on current proposals to improve equity, and how yours is complementary (or not).

***Response***: Thank you for your suggestion. We have addressed this suggestion on additional strategies being employed to improve equity in the introduction. Please see lines 82-84.

Textual comments

Introduction:

64: More than just Canada! The authors argue that vaccine nationalism was a key factor here. This is generally accepted, but you might consider adding a box briefly summarizing/citing a few cases so Canada seems less exceptional

***Response***: We agree and have included additional examples within the text. Please see lines 63-65 and lines 413-416.

65-7: This argument would be strengthened by making explicit reference/explanation to key mechanisms here – for example, the global intellectual property regime.

***Response***: We have included this suggestion, please see lines 72-74.

Methods:

87 -93: I think this is possibly missing the last step. You identified capacity, identified small countries, and then….

***Response***: We have clarified this paragraph, please see lines 99-102.

103-10: Please specify in the Appendix search terms, databases used, dates and languages search, which pharmaceutical companies and international organizations, how news websites were searched etc. It doesn’t necessarily need to be a full formalized PRISMA-style scoping review but it needs to have enough basic information to be replicable.

***Response***: Thank you for your comment and we agree with Reviewer 2 that it is important to include methodological information for reproducibility. To this end we have included the detailed methodology in the Supporting Information file and lines 118-120 in the main manuscript file.

111-112: I got confused because above it says small countries (5) and then very small (15), but here just small. Remind the reader of both.

***Response***: We have clarified this point. Please see lines 122-124.

112: One documented manufacturing facility, as identified from the lit review above? Or a single source/database?

***Response***: We have clarified this sentence. Please see lines 124-127.

Results:

127: “with vaccine manufacturing hubs” – it’s just any vaccine manufacturing right? Not hubs per se?

***Response***: Thank you for pointing this out, we have re-written the sentence for clarity. Please see line 158.

Fig 2 / methods: 6/12 countries identified are in the European Union, where export controls were (to my knowledge) largely at the EU and not sub-national level. It would be good to address why (or why not) you are still including small countries within the EU.

***Response***: Please see response above and lines 413-424 in the Discussion section.

Nice S1 Appendix table.

***Response***: Thank you very much for your feedback.

164-65: Is this a typo? S2 looks likes it is assembled by company, not country.

***Response***: Thank you for bringing this up and apologies for the confusion. In this sentence, we are referring to the S2 Table in the Supporting Information file and not the S1 Appendix table. The S2 Table is assembled by country.

S3: Why are some products in parentheses but not others in “manufacturing countries”?

***Response***: Thank you for pointing this out. Some vaccines are in parenthesis in the “manufacturing countries” column as they have been assigned specific names in respective countries – for example, the Oxford/AstraZeneca vaccine was known as CoviShield in India. We have clarified this point, please see S3 Table in the Supporting Information file.

196-201: This is almost word for word / the sentences are only very lightly rearranged from the WHO page. I noticed because I looked up the WHO page to double check something. Cite / add direct quotes where appropriate.

***Response***: Thank you for your comment. We have re-written the sentence and cited WHO’s prequalification page. Please see lines 128-133.

203: cite

***Response***: Thank you for pointing this out. We have included the citation. Please see line 136.

196-212: Not really results – perhaps should be put further up and integrated within methods as this is essentially a subgroup analysis.

***Response***: We agree and have moved this paragraph to the methods. Please see lines 128-145.

Discussion:

246-7: confusing wording “imposing maximums for both based on real time production capacity or requiring public health justification”. Imposing maximum export controls? As in number of controls?

***Response***: We have clarified this sentence. Please see lines 259-264.

247-251: As a reader I’m losing track of latter… is former/latter APAs/export controls, or is it prohibition/limits?

***Response***: We have clarified this sentence. Please see lines 259-264.

254: The ongoing Pandemic Treaty negotiations (worth specifying, as there are quite a few salient policy processes right now)

***Response***: We have clarified this sentence. Please see line 268.
